# Improving Vector Evaluated Particle Swarm Optimisation by Incorporating Nondominated Solutions

**DOI:** 10.1155/2013/510763

**Published:** 2013-05-07

**Authors:** Kian Sheng Lim, Zuwairie Ibrahim, Salinda Buyamin, Anita Ahmad, Faradila Naim, Kamarul Hawari Ghazali, Norrima Mokhtar

**Affiliations:** ^1^Faculty of Electrical Engineering, Universiti Teknologi Malaysia, 81310 Johor Bahru, Malaysia; ^2^Faculty of Electrical and Electronic Engineering, Universiti Malaysia Pahang, 26600 Pekan, Malaysia; ^3^Department of Electrical Engineering, Faculty of Engineering, University of Malaya, 50603 Kuala Lumpur, Malaysia

## Abstract

The Vector Evaluated Particle Swarm Optimisation algorithm is widely used to solve multiobjective optimisation problems. This algorithm optimises one objective using a swarm of particles where their movements are guided by the best solution found by another swarm. However, the best solution of a swarm is only updated when a newly generated solution has better fitness than the best solution at the objective function optimised by that swarm, yielding poor solutions for the multiobjective optimisation problems. Thus, an improved Vector Evaluated Particle Swarm Optimisation algorithm is introduced by incorporating the nondominated solutions as the guidance for a swarm rather than using the best solution from another swarm. In this paper, the performance of improved Vector Evaluated Particle Swarm Optimisation algorithm is investigated using performance measures such as the number of nondominated solutions found, the generational distance, the spread, and the hypervolume. The results suggest that the improved Vector Evaluated Particle Swarm Optimisation algorithm has impressive performance compared with the conventional Vector Evaluated Particle Swarm Optimisation algorithm.

## 1. Introduction

In multiobjective optimisation (MOO) problems, multiple objective functions are solved simultaneously by either minimising or maximising the fitness of the functions. These multiple objective functions usually conflict with each other. Therefore, the solution to an MOO problem is a set of multiple tradeoffs, or nondominated solutions, rather than a single solution.

The Vector Evaluated Particle Swarm Optimisation (VEPSO) [1] algorithm introduced by Parsopoulos and Vrahatis has been used to solve various MOO problems, such as the design of radiometer array antennas [2], the design of supersonic ejectors for hydrogen fuel cells [3], the design of composite structures [4], the design of steady-state performance for power systems [5], and the design of multiple machine-scheduling systems [6]. In the VEPSO algorithm, one swarm of particles optimises an objective function using guidance from the best solution found by another swarm.

The nondominated solutions found during the optimisation are usually preferred for effective guidance [7]. As an example, the multiobjective PSO (MOPSO) algorithm [8, 9] divides all nondominated solutions into several groups based on their locations in the objective space. Then, one of the nondominated solutions is randomly selected from the group that has the fewest solutions to be used as the particle guide. Furthermore, the nondominated sorting PSO (NSPSO) algorithm [10] uses the primary mechanism of nondominated sorting genetic algorithm-II [11], in which one nondominated solution is randomly selected to be used as the guide for the particles based on the niche count and nearest-neighbour density estimator. In addition, the optimised MOPSO (OMOPSO) algorithm [12] by Margarita Reyes-Sierra and Carlos Coello Coello uses the crowding distance mechanism for binary tournaments to select one of the nondominated solutions as the guide for each particle. Abido [13] uses two nondominated solutions, a local set and a global set, to optimise the problem. Each particle is guided by the nondominated solution that has smallest distance between the particle and both nondominated solution sets.

The conventional VEPSO algorithm solves an MOO problem by improving the solutions in a swarm under the guidance of the best solution with respect to a single objective, found by another swarm. However, the nondominated solution which has better fitness with respect to the other objectives may exist, but it was not used to guide the particles in other swarm. The nondominated solutions are always equal or better solutions compared with the best solution used in conventional VEPSO. The superiority of the nondominated solutions motivates the use of nondominated solutions as particle guides for each swarm in improving the VEPSO algorithm. Thus, in this study, the guidance of a swarm is selected by the nondominated solution which has best fitness with respect to a single objective function, optimised by the other swarm.

The paper is organized as follows. In Section 2, we explain some information on MOO problem. Then, in Section 3, we explain the particle swarm optimisation (PSO), the conventional VEPSO, and the improved VEPSO algorithms. In the next section, we demonstrate the simulation experiment which includes several performance measures and benchmark test problem, before we discuss the results. Lastly, we present the conclusion and include some suggestion for future work.

## 2. Multiobjective Optimisation

Consider a minimisation of a multiobjective problem:

(1) minimise  the  fitness  function, F→(x→)={fm(x→)∈ℜM,  m=1,2,…,M}  subject  to  gj(x→)≤0, j=1,2,…,J,hk(x→)=0, k=1,2,…,K,

where $\vec{x}=\left\{x_{n}\in {ℜ}^{N},\;n=1,2,\ldots ,N\right\}$ is the *N*-dimensional vector of decision variables that represent the possible solutions, *M* is the number of objectives, *f*_*m*_ ∈ *ℜ* ^*M*^ is the objective function, and {*g*_*j*_, *h*_*k*_} ∈ *ℜ* are the inequality and equality constraint functions, respectively.

**Figure 1:**
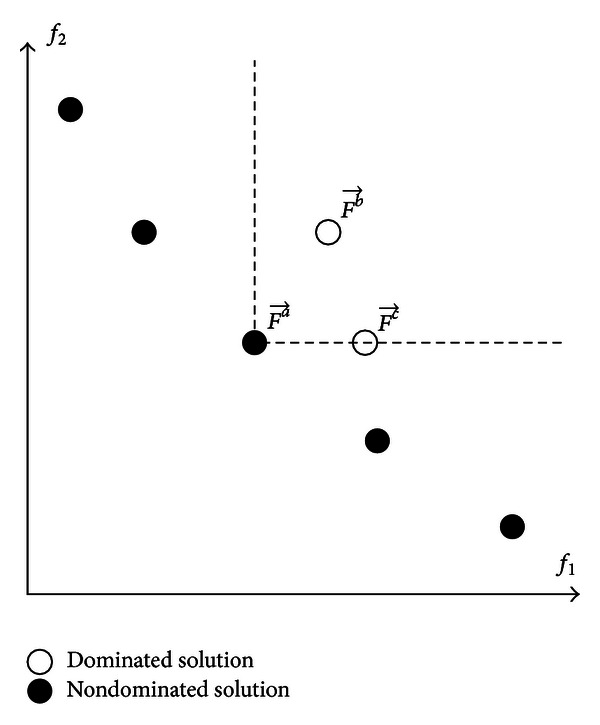
Dominance relation for two objectives problem.

In explaining the concept of Pareto optimality, consider two vectors $\{\vec{F^{a}},\vec{F^{b}}\}\in {ℜ}^{M}$. $\vec{F^{a}}$ *dominates* $\vec{F^{b}}$ (denote as $\vec{F^{a}}≺\vec{F^{b}}$) if and only if *f*_*m*_^*a*^ ≤ *f*_*m*_^*b*^ for *m* = 1,2,…, *M* and *f*_*m*_^*a*^ < *f*_*m*_^*b*^ at least once. The dominance relations $\vec{F^{a}}≺\vec{F^{b}}$ and $\vec{F^{a}}≺\vec{F^{c}}$ for a two-objective problem are indicated by the labelled circles in Figure 1. Hence, a vector of decision variables $\vec{x^{a}}$ is a *nondominated solution* if and only if there is no other solution $\vec{x^{b}}$ such that $\vec{F}(\vec{x^{a}}\;)≺\vec{F}(\vec{x^{b}}\;)$. The nondominated solution is also known as the Pareto optimal solution.  The set of nondominated solutions of an MOO problem is known as the *Pareto Optimal set*,  *𝒫*. The set of objective vectors with respect to *𝒫* is known as the *Pareto Front*, $𝒫ℱ=\{\vec{F}(\vec{x})\in {ℜ}^{M}|\vec{x}\in 𝒫\}$. The *𝒫ℱ* for a two-objective problem is illustrated by the black circles in Figure 1.

The goal of an MOO algorithm is to find as many nondominated solutions as possible according to the objective functions and constraints. The Pareto front corresponding to the nondominated set should be as close to and well distributed over the true Pareto front as possible. However, it is possible to have different solutions that map to the same fitness value in objective space. 

## 3. Particle Swarm Optimisation

### 3.1. Original Particle Swarm Optimisation Algorithm

 Based on the social behaviour of birds flocking and fish schooling, a population-based stochastic optimisation algorithm named Particle Swarm Optimisation (PSO) was introduced by Kennedy et al. [14, 15]. The PSO algorithm contains individuals referred to as particles that encode the possible solutions to the optimisation problem using their positions. These particles explore the defined search space to look for solutions that better satisfy the objective function of the optimised problem. Each particle collaborates with the others during the search process by comparing its current position with the best position that it and the other particles in the swarm have found [16].

**Figure 2:**
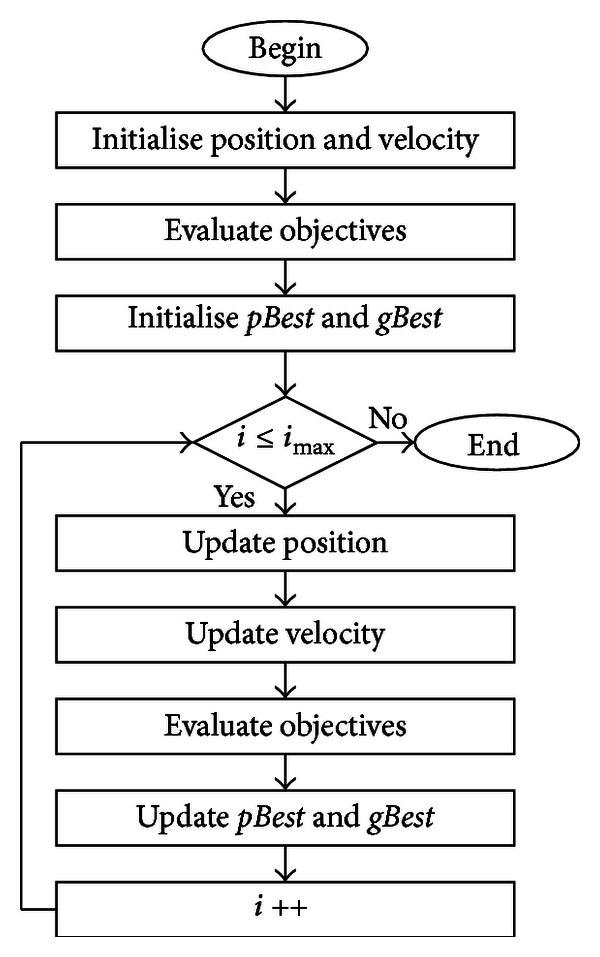
The PSO algorithm.

Figure 2 shows the flow chart of the PSO algorithm. For the PSO algorithm, consider the following minimisation problem: there are *I*-particles flying around in an *N*-dimensional search space, where their positions, *p*_*n*_^*i*^  (*i* = 1,2,…, *I*; *n* = 1,2,…, *N*), represent the possible solutions. Initially, all particles are randomly positioned in the search space and assigned random velocities, *v*_*n*_^*i*^(*t*). Then, the objective fitness, $\vec{F^{i}}(t)$, for each particle is evaluated by calculating the objective functions with respect to *p* ^*i*^(*t*). Next, each particle's best position, *pBest* ^*i*^(*t*), is initialised to its current position. Meanwhile, the best among all *pBest* ^*i*^(*t*) is set as the swarm's best position, *gBest*(*t*), as specified in (2), where *S* is the swarm of particles:

(2) $$\begin{array}{c} gBest=\left\{pBest^{i}\in S\;|\;f\left(pBest^{i}\right)=\min f\left(\forall pBest^{i}\in S\right)\right\}.\; \end{array}$$

Next, the algorithm iterates until the stopping condition is met; that is, either the maximum number of iterations is exceeded or the minimum error is attained. In each iteration, each particle's velocity and position are updated using (3) and (4), respectively,

(3) vni(t+1)=χ[ωvni(t)+c1r1(pBestni−pni(t))+c2r2(gBestn−pni(t))],

(4) $$\begin{array}{c} p_{n}^{i}\left(t+1\right)=p_{n}^{i}\left(t\right)+v_{n}^{i}\left(t+1\right), \end{array}$$

where *χ* is the constriction factor and *ω* is the inertia weight. *c*_1_ and *c*_2_ are the cognitive and social coefficients, respectively. Meanwhile, *r*_1_ and *r*_2_ are both random values between zero and one. After the velocity and position are updated, the $\vec{F^{i}}(t)$ for each particle is evaluated again. Later, *pBest* ^*i*^(*t*) is updated with the more optimal between the new position of the *i*th particle or *pBest* ^*i*^(*t*). Then, the *gBest*(*t*) is updated with the most optimal *pBest* ^*i*^(*t*) among all the particles, as given in (2). Finally, when the stopping condition is met, *gBest*(*t*) represents the optimum solution found for the problem optimised using this algorithm.

### 3.2. Vector Evaluated Particle Swarm Optimisation Algorithm

 Parsop$\overset{´}{\text{o}}$ulos and Vrahatis [1] introduced the VEPSO algorithm, which was inspired by the multiswarm concept of the VEGA algorithm [17]. In this multiswarm concept, each objective function is optimised by a swarm of particles using the *gBest*(*t*) from another swarm. The *gBest*(*t*) for the *m*th swarm is the *pBest* ^*i*^(*t*) that has most optimal fitness with respect to the *m*th objective, among all *pBest* ^*i*^(*t*) from the *m*th swarm, as given below:

(5) gBestm={pBesti∈Sm ∣ fm(pBesti)=min⁡fm(∀pBesti∈Sm)}.

Generally, the PSO and VEPSO algorithms have similar process flows, except that all processes are repeated for *M* swarms when optimising problems with *M* objective functions. Because each swarm optimises using *gBest*(*t*) from another swarm, in VEPSO, the velocity is updated using (6). The velocity equation for particles in the *m*th swarm updates *gBest* ^*k*^(*t*), where *k* is given in (7):

(6) vnmi(t+1)=χ[ωvnmi(t)+c1r1(pBestnmi−pnmi(t))+c2r2(gBestnk−pnmi(t))],

(7) $$\begin{array}{c} k=\{\begin{array}{cc} M, & m=1,\\ m-1, & \text{otherwise}. \end{array} \end{array}$$

In addition to the difference in the velocity equation, all nondominated solutions found during the optimisation are stored in an archive each time after the objective functions are evaluated. To ensure that the archive contains nondominated solutions only, the fitness $\vec{F^{i}}(t)$ of each particle is compared, based on the Pareto optimality criterion, to those of all particles before it is compared to the nondominated solutions in the archive. All nondominated solutions in the archive represent possible solutions to the MOO problem.

### 3.3. The Improved VEPSO Algorithm

**Figure 3:**
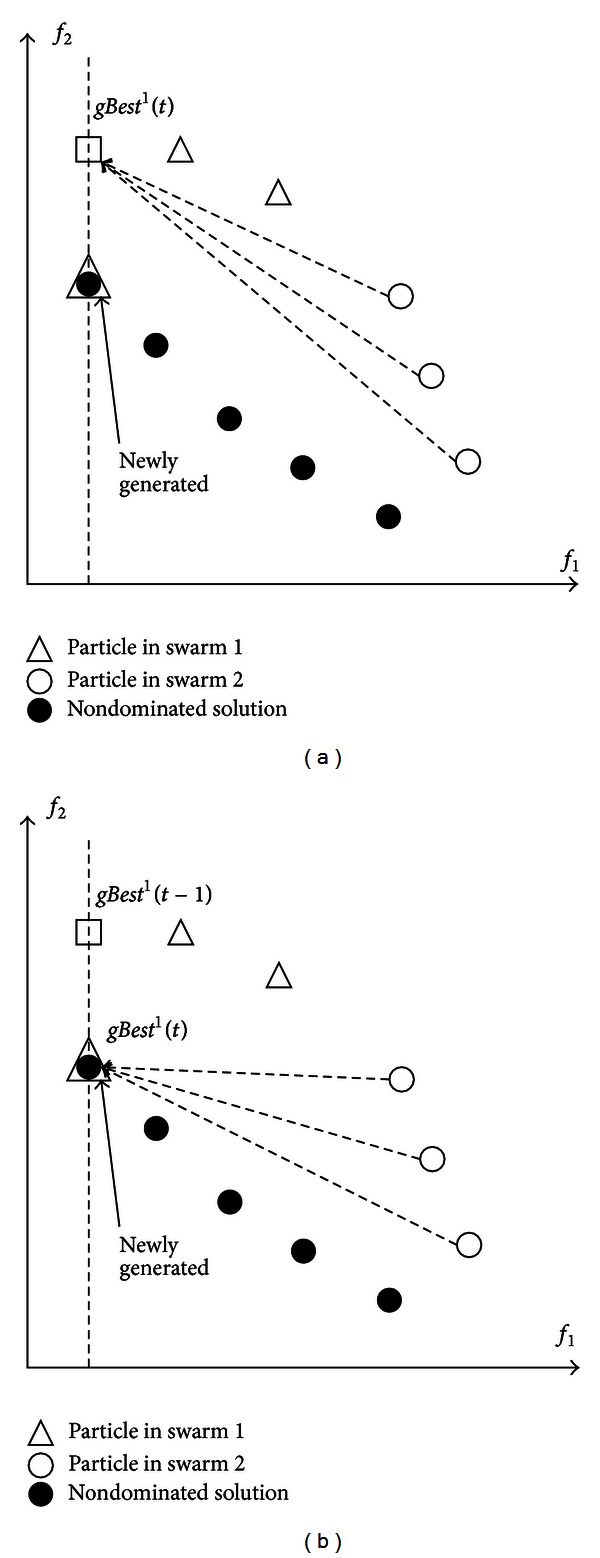
Particles guided by (a) the best solution from the other swarm and (b) a nondominated solution.

In conventional VEPSO, each particle of a swarm is updated by the *gBest*(*t*) from the other swarm that is optimal with respect to the objective function optimised by the other swarm. Consider a two-objective optimisation problem as an example; the *gBest*(*t*) of the first swarm is only updated when a newly generated solution has better fitness with respect to the first objective, as specified in (5). Thus, *gBest*(*t*) is not updated even if the new solution, nondominated solution, has equal fitness with respect to the first objective and better fitness with respect to the second objective. Hence, as in Figure 3(a), each particle from the second swarm moves under the guidance of the *gBest* ^1^(*t*) but not the better, nondominated solutions.

However, this limitation can be overcome by updating *gBest*(*t*) with a new solution, nondominated solution, that has equal fitness with respect to the optimised objective function and better fitness with respect to the other objective. This improved VEPSO algorithm is represented in Figure 3(b), where *gBest* ^1^(*t*) is now a nondominated solution that is best with respect to the first objective function. Thus, each particle from the second swarm will be guided by its own *pBest* ^2^ *i*(*t*) and *gBest* ^1^(*t*), which is a nondominated solution, with the hope that the particle will converge toward the Pareto front faster.

In the improved VEPSO algorithm, the generality of conventional VEPSO is not lost; so the *gBest*(*t*) of a swarm is the best nondominated solution with respect to the objective function optimised by the swarm. Therefore, the *gBest*(*t*) of the *m*th swarm is given as following:

(8) $$\begin{array}{c} gBest^{m}=\left\{X\in 𝒫\;|\;f_{m}\left(X\right)=\min f_{m}\;\;\left(\forall X\in 𝒫\right)\right\}, \end{array}$$

where *X* is a nondominated solution and *𝒫* is the set of nondominated solutions in the archive. For a two-objective-function problem, the particles from the second swarm are guided by the nondominated solution that is best with respect to the first objective function. Meanwhile, the particles of the first swarm are guided by the nondominated solution that is optimal with respect to the second objective function. Thus, this improved algorithm is called Vector Evaluated Particle Swarm Optimisation incorporate nondominated solutions (VEPSOnds).

In addition, the PSO algorithm has the natural limitation that particles tend to become stuck in locally optimal solutions [18, 19]. Therefore, this improved VEPSO algorithm also includes the polynomial mutation mechanism from nondominated sorting genetic algorithm-II [11]. The polynomial mutation mechanism modifies the particle position with a certain probability such that the particle can mutate out from the locally optimal solution and continue the search for a globally optimal solution. In this work, one of every ten particles is mutated in the improved VEPSO algorithm.

## 4. Experiment

### 4.1. Performance Measure

In order to analyse the performance of the VEPSOml algorithm, several quantitative performance mesasures are used. Since MOO problems have different features, for example multilocal optima solution, which could trap the particles from obtaining more nondominated solutions; hence, the number of solution (NS) measure is used to quantify the total number of nondominated solutions found at the end of the computation. Besides, for example when the particles one trapped in a local optima solution, the obtained Pareto front will not be converged close to the true Pareto front which means that the best possible solutions were not found yet. Thus, the generational distance (GD) [20] is used and defined as the average Euclidean distance between the obtained Pareto front, *𝒫ℱ*_*o*_, and the true Pareto front, *𝒫ℱ*_*t*_, using (9). A smaller GD value indicates better performance:

(9) $$\begin{array}{c} \text{GD}=\frac{{\left(\sum_{q=1}^{||𝒫{ℱ}_{o}||}d_{q}^{M}\right)}^{1/M}}{||𝒫{ℱ}_{o}||},\\ d_{q}=\underset{1\leq k\leq ||𝒫{ℱ}_{t}||}{\min}\sqrt{\sum_{j=1}^{M}{\left(f_{j}^{q}-f_{j}^{k}\right)}^{2}.} \end{array}$$

**Figure 4:**
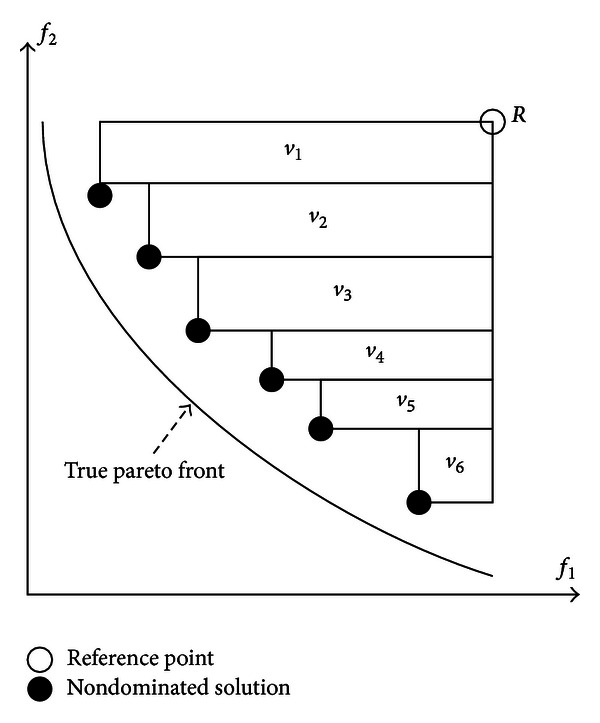
Hypervolume measure with area covered by the nondominated solutions and a reference point.

A well-converged Pareto front does not guarantee to have good diversity of nondominated solutions along the Pareto front. Therefore, the third performance metric used is the spread (SP) [11], which is used to measure the extent of the distribution of the *𝒫ℱ*_*o*_ along the *𝒫ℱ*_*t*_. Equations (10), are used to measure SP, and smaller values indicate better performance:

(10) $$\begin{array}{c} \text{SP}=\frac{d_{f}+d_{l}+\sum_{q=1}^{||𝒫{ℱ}_{o}||-1}\left|d_{q}-\overset{-}{d}\right|}{d_{f}+d_{l}+\left(||𝒫{ℱ}_{o}||-1\right)\overset{-}{d}},\\ \overset{-}{d}=\frac{\sum_{q=1}^{||𝒫{ℱ}_{o}||-1}d_{q}}{||𝒫{ℱ}_{o}||-1},\\ d_{q}=\sqrt{{\left(f_{1}^{q}-f_{1}^{q+1}\right)}^{2}+{\left(f_{2}^{q}-f_{2}^{q+1}\right)}^{2}}, \end{array}$$

where *d*_*f*_ is the Euclidean distance between the first extreme members in *𝒫ℱ*_*o*_ and *𝒫ℱ*_*t*_ and *d*_*l*_ is the Euclidean distance between the last extreme members in *𝒫ℱ*_*o*_ and *𝒫ℱ*_*t*_. In some cases, the obtained Pareto fronts could be converged well to the true Pareto front but it has poor diversity performance. Hence, it is not fair by comparing different algorithms with the GD and SP measures only. Finally, the hypervolume (HV) [21] is used to measure the total space or area enclosed by the *𝒫ℱ*_*o*_ and a reference point, *R*, which is a vector constructed from the worst objective value from the *𝒫ℱ*_*t*_. Equation (11) is used to evaluate the HV value. The total area for HV is the enclosed area in Figure 4 and is calculated using (11). Larger HV values represent better performance:

(11) $$\begin{array}{c} \text{HV}=\sum_{q=1}^{||𝒫{ℱ}_{o}||}v_{q}, \end{array}$$

where *v*_*q*_ is the space or area between *R* and the diagonal corner of *q*th solution of *𝒫ℱ*_*o*_.

### 4.2. Test Problems

Five of the benchmark test problems from ZDT [22] are used to evaluate the performance of the algorithm. Because this study focused on continuous search space problems, the ZDT5 problem is not used as it is for the evaluation of binary problems. All benchmark problems are set up using the parameter values recommended in the paper [22]. For evaluating the performance measure, the true Pareto front for each problem is obtained from the standard database generated by the jMetal (http://jmetal.sourceforge.net/problems.html).

### 4.3. Evaluation of VEPSO Algorithms

Because the VEPSOnds algorithm includes polynomial mutation, the experiment in this work should analyse a version of VEPSOnds that does not include polynomial mutation. This implementation exists because the polynomial mutation affects the algorithm's performance, and it is necessary to determine whether the change in performance is due to the use of multiple nondominated solutions or the polynomial mutation. Thus, in this work, the VEPSOnds algorithm without mutation is denoted as VEPSOnds1 and the VEPSOnds algorithm with mutation is denoted as VEPSOnds2.

In this experiment, the total number of particles is fixed to 100 and divided equally among all swarms. The archive size is controlled by removing the nondominated solutions with the smallest crowding distance [11]. In addition, the maximum iteration and archive size are set to 250 and 100, respectively. During the computation, the inertia weight is linearly degraded from 1.0 to 0.4. The cognitive and social constants are both random values between 1.5 and 2.5. Moreover, the distribution index is set to 0.5 for the mutation operation. Each test problem is simulated for 100 runs to enable statistical analysis.

The performance of each algorithm tested on the ZDT1 problem is presented in Table 1. For the average NS measure, the number of nondominated solutions found by both improved VEPSO algorithms was significantly greater for conventional VEPSO. For the GD measure, VEPSOnds1 demonstrated significant improvement compared with the conventional VEPSO algorithm. Meanwhile, the VEPSOnds2 algorithm exhibited an extremely large improvement compared with both conventional VEPSO and VEPSOnds1. Similarly, the SP measures for both improved VEPSO algorithms also indicated significant improvement compared with conventional VEPSO. As expected, the HV performance also improved dramatically when the problem was optimised using multiple nondominated solutions as particle guides.

**Figure 5:**
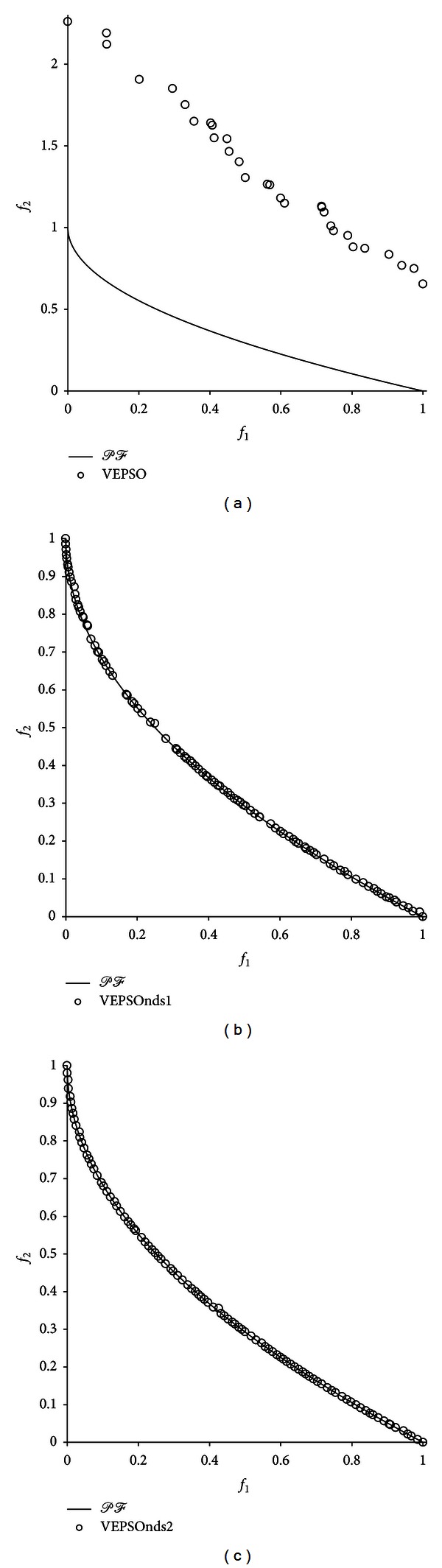
Plot of nondominated solutions returned by each algorithm for the ZDT1 test problem.

For better visual comparison, the Pareto fronts with the best GD value returned for each test problem are shown in Figure 5 through Figure 9. Figure 5 shows the plot of nondominated solutions with the best GD measure returned for the ZDT1 problem. The nondominated solutions obtained by VEPSO are clearly located very far away from the true Pareto front, which leads to a large GD value. Moreover, the obtained solutions are unevenly distributed around the objective space, which yields a large SP value. In contrast, the VEPSOnds1 and VEPSOnds2 algorithms generated nondominated solutions close to and evenly distributed over the true the Pareto front. Therefore, the GD and SP values for both improved VEPSO algorithms are significantly smaller than those for conventional VEPSO. However, the VEPSOnds2 has better distribution of nondominated solutions than the VEPSOnds1.

Table 2 presents the performance measures for all algorithms tested on the ZDT2 problem. Again, both the improved VEPSO algorithms dramatically improved the ability to obtain a large number of solutions compared with VEPSO, especially VPESOnds2. In addition to the NS performance, the GD and SP performances were also dramatically improved because the nondominated solutions used in the improved VEPSO algorithms are better guides compared with the best solution among each particle, which is used in conventional VEPSO. However, in SP measure, the VEPSOnds1 shows negligible improvement, whereas the VEPSOnds2 shows distinguished improvement over the conventional VEPSO. The conventional VEPSO algorithm was unable to yield a meaningful HV because the obtained nondominated solutions were far worse than the true Pareto front. However, the VEPSOnds1 and VEPSOnds2 algorithms yielded good HV values. 

**Figure 6:**
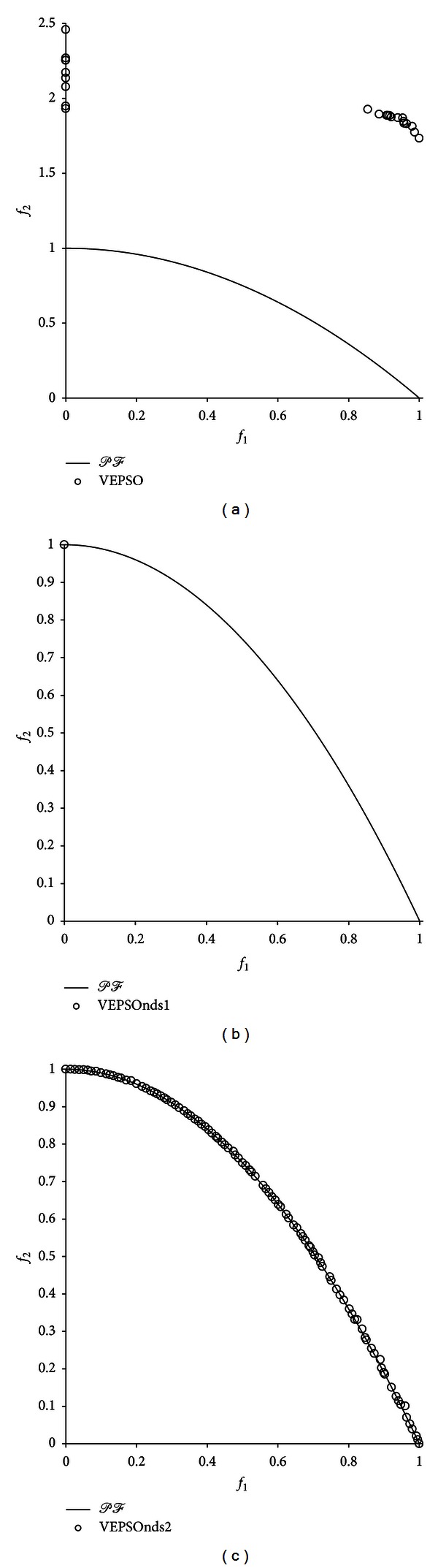
Plot of nondominated solutions returned by each algorithm for the ZDT2 test problem.

Figure 6 shows the nondominated solutions with the best GD measure returned for the ZDT2 problem. The poor performance of conventional VEPSO is visible because the nondominated solutions found are very distant from the true Pareto front and distributed unevenly in the objective space. Conversely, the VEPSOnds1 algorithm was able to obtain a nondominated solution that is located on the true Pareto front. However, there is only one nondominated solution, which increases the SP value of this algorithm. In contrast, the VEPSOnds2 algorithm successfully found nondominated solutions very close to the true Pareto front, and the nondominated solutions found are distributed evenly over the true Pareto front. Thus, the polynomial mutation preventing the particles from converging too early is an important mechanism in improving the diversity performance of the algorithm. 

Table 3 presents the performance measures of all algorithms tested for the ZDT3 problem. Regarding the NS measure, both the improved VEPSO algorithms successfully obtained a large number of nondominated solutions. Moreover, both improved VEPSO algorithms yielded great improvement compared with the conventional VEPSO in terms of convergence. However, the SP value of the solutions obtained by both improved VEPSO was degraded in this test. Even with the degradation in the diversity performance of both improved VEPSO, they still hold the performance advantages with their superior convergence improvement. Besides, both improved VEPSO performances are better as their HV value was also improved when the particles in the algorithm used additional guides during the optimisation.

**Figure 7:**
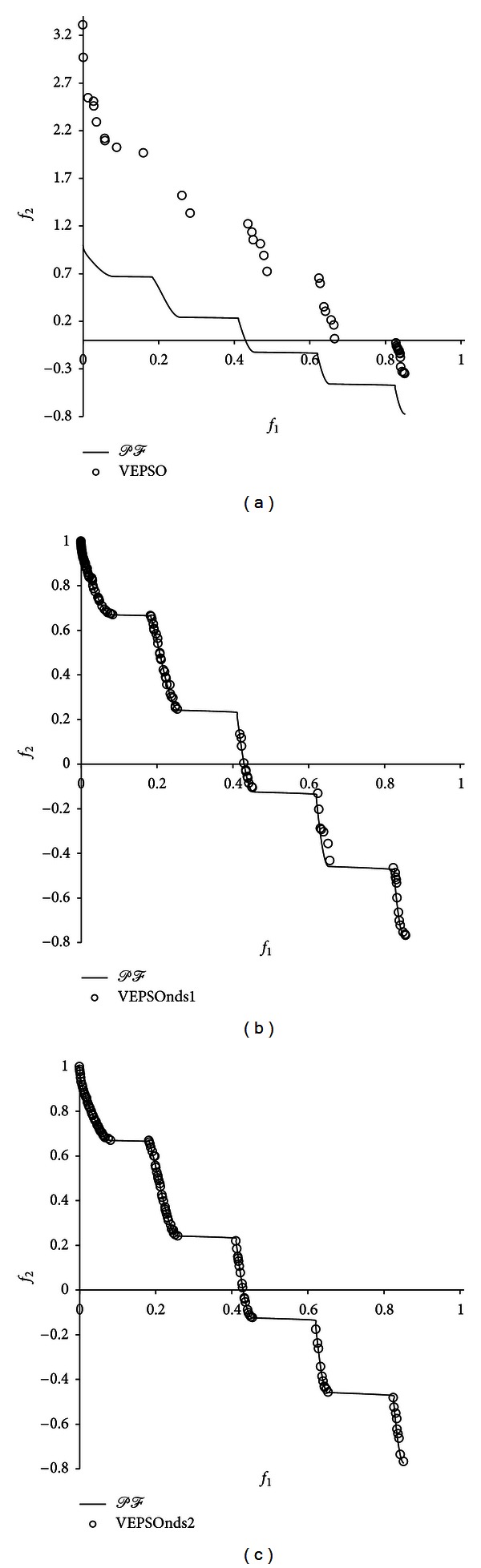
Plot of nondominated solutions returned by each algorithm for the ZDT3 test problem.

Figure 7 shows the nondominated solutions with the best GD measure returned for ZDT3 problem. Unavoidably, the nondominated solutions obtained by the conventional VEPSO algorithm are scattered far from the true Pareto front, which leads to poor performance. Conversely, both the improved VEPSO algorithms were able to obtain nondominated solutions that cover the true Pareto front almost perfectly. Hence, both the improved VEPSO algorithms exhibited almost equal improvement, but VEPSOnds1 has weaker diversity performance as there are lesser solutions at the middle of the Pareto front.

Table 4 presents the performance measures for all algorithms tested for the ZDT4 problem. The average number of nondominated solutions found by the conventional VEPSO algorithm is relatively low compared with VEPSOnds1, which found most of the solutions. The conventional VEPSO algorithm had great difficulty escaping from the multiple local optima, which resulted in a very large GD value. However, the improved VEPSO algorithms, in which particles are guided by the nondominated solutions, had less chance of being stuck in local optima. Meanwhile, the HV value yielded by the conventional VEPSO algorithm is relatively small compared with that of the improved VEPSO algorithms. Thus, the smaller SP value for conventional VEPSO does not mean it has better performance, as both improved VEPSO still maintain performance advantages with their better GD and HV values.

**Figure 8:**
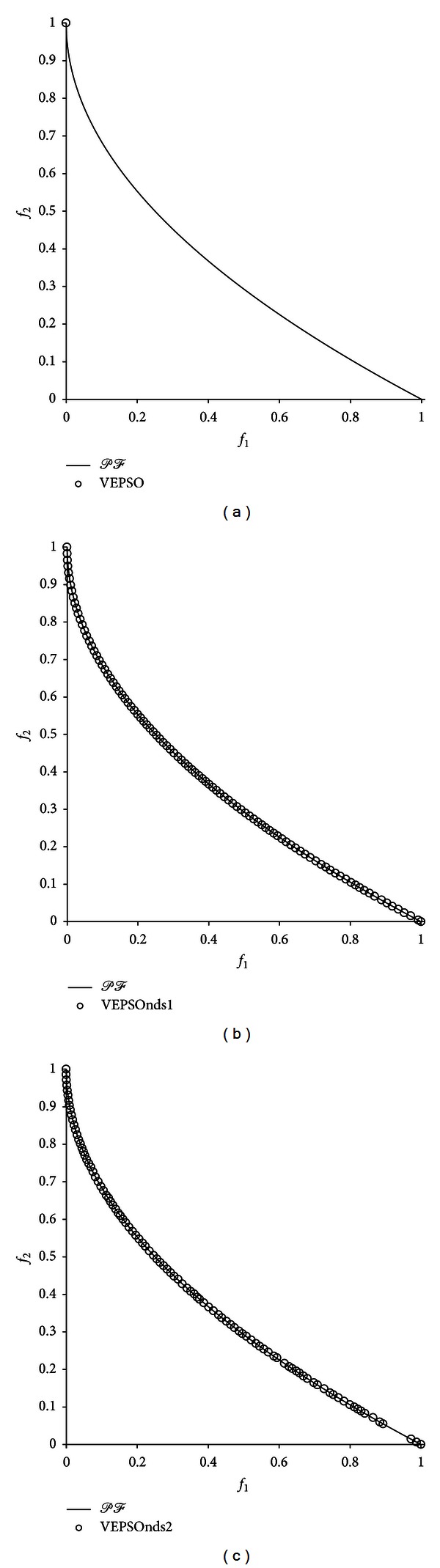
Plot of nondominated solutions returned by each algorithm for the ZDT4 test problem.

Figure 8 shows the nondominated solutions with the best GD measure returned for the ZDT4 problem. The conventional VEPSO algorithm, in which particles follow only one guide, was easily stuck in local optima, as shown in the first plot. Thus, the algorithm was able to find only one nondominated solution. However, both the improved VEPSO algorithms, in which additional guides are used, had less difficulty in obtaining a greater number of diverse nondominated solutions.

Table 5 presents the performance measures for all algorithms tested on the ZDT6 problem. Interestingly, all algorithms found approximately the same number of nondominated solutions. Moreover, the SP and HV values for all algorithms are also similar. However, noticeably, both improved VEPSO have outperformed the conventional VEPSO in terms of convergence performance.

**Figure 9:**
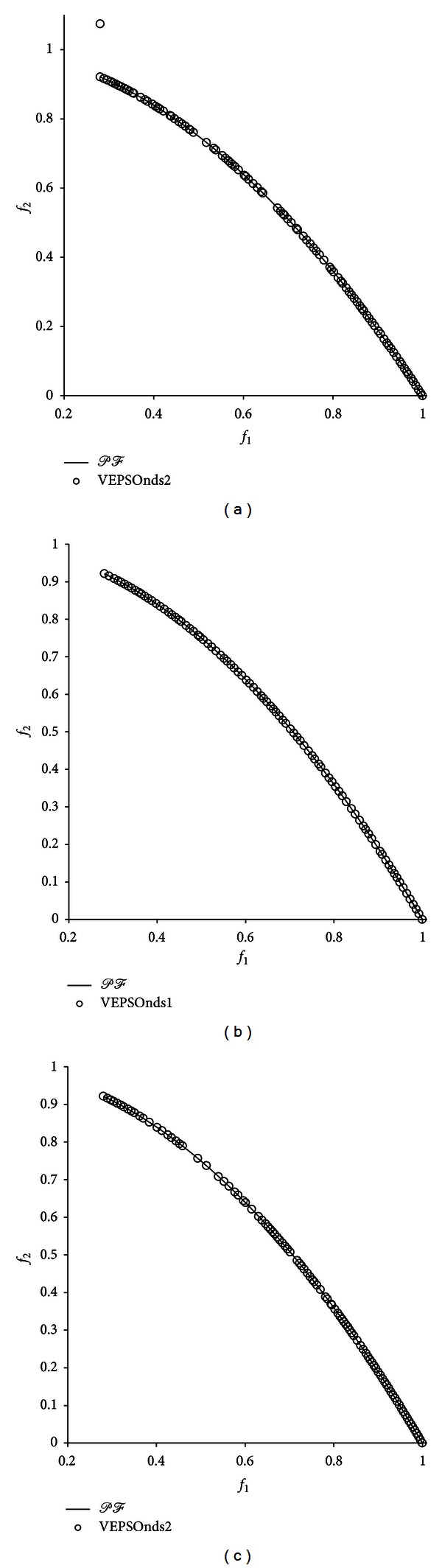
Plot of nondominated solutions returned by each algorithm for the ZDT6 test problem.

Figure 9 shows the nondominated solutions with the best GD measure returned for the ZDT6 problem. As predicted, the plots of nondominated solutions are similar because all algorithms exhibit similar results in terms of convergence and diversity. However, the nondominated solutions for the VEPSOnds2 algorithm were not well distributed over the true Pareto front, middle of the Pareto front in this case, which caused the algorithm to have the largest SP value, as shown in Table 5.

For all test problems, the improved VEPSO algorithms exhibited significant improvement compared with the conventional VEPSO algorithm for most of the performance measures. The performance improvements occurred because the nondominated solutions always provide a better solution than a solution that optimises only a single-objective function. Using a better solution as the leader increases the quality of the result.

### 4.4. Analysis of the Number of Particles

**Figure 10:**
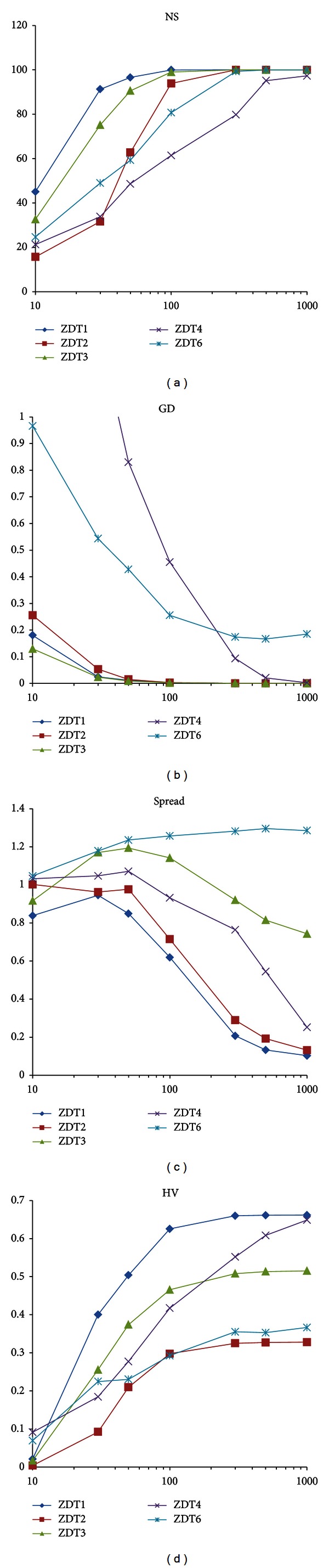
Plots of the performance metrics for various numbers of particles. (a) Number of solutions. (b) Generational distance. (c) Spread. (d) Hypervolume.

The performance of the VEPSOnds2 algorithm with various numbers of particles is analysed in this experiment. Most of the parameters are the same as in the previous experiment, except that the particles are equally divided between swarms for a total of 10, 30, 50, 100, 300, 500, and 1000 particles. The performance measurements, taken for each total number of particles and for each benchmark problem, are plotted in Figure 10.

In short, the performance of VEPSOnds2 improves when the number of particles is increased. When VEPSOnds2 is computed with 250 iterations, the algorithm performs well at 300 particles, which is equivalent to 75 000 function evaluations. With a higher number of particles, the algorithm exhibits even better results, but the computational time increases dramatically.

### 4.5. Analysis of the Number of Iterations

**Figure 11:**
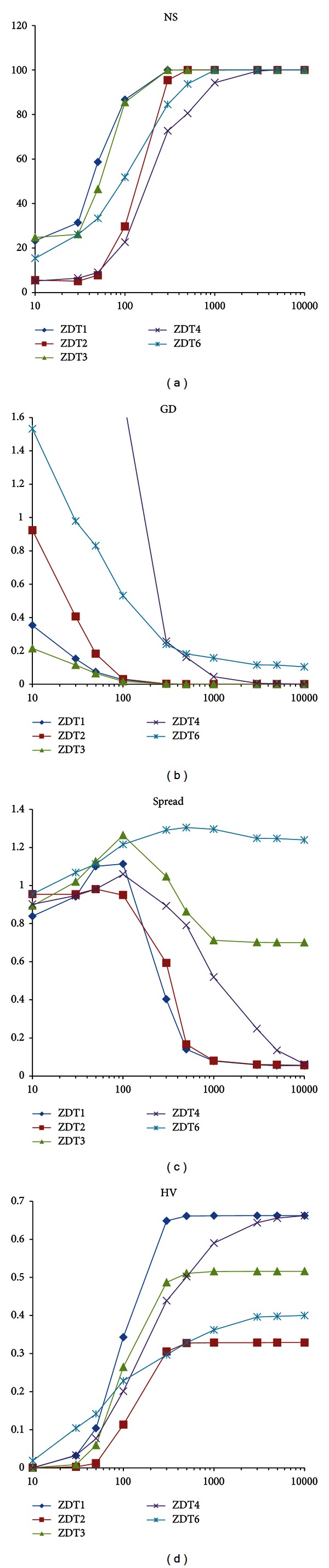
Plots of the performance metrics for various numbers of iterations. (a) Number of solution. (b) Generational distance. (c) Spread. (d) Hypervolume.

The effect of various numbers of iterations on VEPSOnds2 performance is investigated in this experiment. In this experiment, the number of iterations becomes 10, 30, 50, 100, 300, 500, 1000, 3000, 5000, or 10 000. All parameters are set as in the previous experiments, and the number of particles is set to 100, which is divided equally between swarms. The plot of performance metrics for the various numbers of iterations for each benchmark problem is displayed in Figure 11.

When the number of iterations is increased, the performance of VEPSOnds2 improves. The VEPSOnds2 algorithm performs consistently and acceptably with 100 particles when there are 300 iterations or 30 000 function evaluations. Computation of the algorithm with a higher number of iterations, such as 3000 particles or 300 000 function evaluations, could result in a better performance but is only recommended if a powerful computing platform is used.

### 4.6. Benchmarking with the State-of-the-Art Multiobjective Optimisation Algorithms

The VEPSOnds2 algorithm performed better than the other algorithms in most test cases. Thus, the performance of this algorithm is compared to four other MOO algorithms which are nondominated sorting genetic algorithm-II (NSGA-II) [11], strength pareto evolutionary algorithm 2 (SPEA2) [23], archive-based hYbrid scatter search (AbYSS) [24], and speed-constrained multiobjective PSO (SMPSO) [25]. For a fair comparison, all algorithms compute 25 000 function evaluations with the archive size set to 100. The NSGA-II, commonly used for performing comparisons, was set to use a population size of 100 for optimisation. This algorithm was set to use the Simulated Binary Crossover (SBX) operator with the crossover probability *p*_*c*_ = 0.9 and polynomial mutation [26] operators with the mutation probability *p*_*m*_ = 1/*N*. The distribution index for both operators was set to *μ*_*n*_ = *μ*_*m*_ = 20. The SPEA2 was set to use the same parameters as in NSGA-II. The AbYSS was set to use a population size of 20. The pairwise combination parameters in AbYSS were set to RefSet_1_ = 10 and RefSet_2_ = 10. The polynomial mutation parameters were set to similar values as those in NSGA-II and SPEA2. In SMPSO, the population size and maximum iteration were set to 100 and 250, respectively. The terms *r*_1_ = *r*_2_ = random[0.1, 0.5], and the terms *c*_1_ = *c*_2_ = random[1.5, 2.0]. This algorithm was also set to use polynomial mutation [27] with *p*_*m*_ = 1/*N* and *μ*_*m*_ = 20.

Table 6 lists the performance of the algorithms on the ZDT1 test problem. The number of solutions found by the VEPSOnds2 is comparable to the other algorithms. However, the average GD value of the VEPSOnds2 is at least 10 times greater than that of the others even though its minimum GD value is close to that of the other algorithms. VEPSOnds2 also has the highest average SP value, but its minimum SP is better than that of NSGA-II. The HV value for VEPSOnds2 is similar to that of the other algorithms.

Table 7 lists the performance of the algorithms on the ZDT2 test problem. VEPSOnds2 was able to obtain a reasonable number of solutions compared to the other algorithms. However, the GD value for VEPSOnds2 is the highest among all algorithms. Additionally, VEPSOnds2 has the greatest average SP value, even though its minimum SP value is better than that of NSGA-II. In the HV measure, the average value returned by VEPSOnds2 is relatively close to the other algorithms and even outperforms the NSGA-II with its maximum value.

Table 8 lists the performance of the algorithms on the ZDT3 test problem. SMPSO and VEPSOnds2 both show poor performance with respect to the maximum number of solutions for all runs. Again, VEPSOnds2 has a 10 times greater GD value compared to the other algorithms. Interestingly, the diversity performance of VEPSOnds2 is very poor, as the average SP value is higher than 1.0. However, the maximum HV value of VEPSOnds2 was not the smallest, and its average is almost as large as the rest.

Table 9 lists the performance of the algorithms on the ZDT4 test problem. The multiple local optima featured in this problem challenged VEPSOnds2 greatly, as the number of solutions obtained is very low. In addition, the convergence and diversity performances were very poor, as the GD and SP values are both very large compared to the other algorithms. The HV value was also poor, as the multiple local optima feature is well known as a natural weakness in PSO-based algorithms [18, 19].

Finally, Table 10 lists the performances of the algorithms on the ZDT6 test problem. On average, VEPSOnds2 does not obtain the highest number of nondominated solutions, but the number is still in an acceptable range. However, the GD value for VEPSOnds2 was too far from the other algorithms. In addition, the SP value for VEPSOnds2 is extremely large compared to the other algorithms, and the average HV value for VEPSOnds2 is smaller than that for the other algorithms. On a positive note, the maximum HV value for VEPSOnds2 improves upon that for AbYSS, NSGA-II, and SPEA2.

The main purpose of this experiment is to present the overall performance of the improved VEPSO algorithm in comparison to state-of-the art algorithms, not to show how it outperforms them. Indeed, the overall performance of the VEPSOnds2 is not better than all the compared algorithms. However, relatively speaking, its performance is still within the acceptable range and is better than some of the other algorithms in certain cases.

## 5. Conclusions

The conventional VEPSO algorithm uses one swarm to optimise one objective function. The optimisation is guided using only one best solution found by another swarm with respect to the objective function optimised by that swarm. In contrast, recent PSO-based MOO algorithms prefer to use the nondominated solutions as the particle guides. Thus, it is possible to modify the VEPSO algorithm such that the particles are guided by nondominated solutions that are optimal at specific objective function. Five ZDT test problems were used to investigate the performance of the improved VEPSO algorithm based on the measures of the number of nondominated solutions found, the Generational Distance, the Spread, and the Hypervolume.

The experimental results show that the improved algorithms were able to obtain better quality Pareto fronts than conventional VEPSO, especially VEPSOnds2, which consistently returned the best convergence and diversity performance. On the other hand, the introduction of polynomial mutation should reduce the chance for a particle to get stuck in local optima, which features greatly in the ZDT4 test problem. However, VEPSOnds2 did not show much improvement compared to VEPSOnds1. This could possibly be due to the choice of the number of particles that are subject to mutation. Hence, the analysis for proper number of particles subject to mutation should be considered in future work. Even so, VEPSOnds2 is relatively better than VEPSOnds1, as confirmed by most of the performance measurements.

In addition, in VEPSOnds2, the particles of a swarm are guided by the same *gBest*(*t*). Thus, there is a greater chance for them to converge prematurely around the *gBest*(*t*) that might represent a locally optimal solution. On the other hand, in SMPSO, each particle will select one of the nondominated solutions by binary tournament, using the crowding distance as its guide. This means that in SMPSO, each particle has a different *gBest*(*t*) as a guide during optimisation. Thus, the future VEPSOnds2 algorithm should reduce the chances for all particles to follow the same *gBest*(*t*), in order to prevent premature convergence.

## Figures and Tables

**Table 1:** Algorithm performance tested on ZDT1 problem.

Measure	VEPSO	VEPSOnds1	VEPSOnds2
NS			
Ave.	30.220000	100.000000	99.790000
SD	5.697031	0.000000	1.458483
Min.	16.000000	100.000000	86.000000
Max.	44.000000	100.000000	100.000000
GD			
Ave.	0.295865	0.022637	0.002194
SD	0.051645	0.014201	0.003505
Min.	0.139491	0.000283	0.000169
Max.	0.432478	0.073477	0.019113
SP			
Ave.	0.834481	0.729350	0.571807
SD	0.039111	0.160298	0.248304
Min.	0.705367	0.322322	0.168144
Max.	0.917087	1.219625	1.127141
HV			
Ave.	0.001886	0.428153	0.631216
SD	0.010058	0.113432	0.046091
Min.	—	0.185313	0.438793
Max.	0.087426	0.659603	0.661363

**Table 2:** Algorithm performance tested on ZDT2 problem.

Measure	VEPSO	VEPSOnds1	VEPSOnds2
NS			
Ave.	8.070000	38.120000	97.490000
SD	6.356822	25.747131	7.832198
Min.	1.000000	1.000000	49.000000
Max.	24.000000	100.000000	100.000000
GD			
Ave.	0.766956	0.039653	0.002003
SD	0.324444	0.063791	0.003483
Min.	0.240509	0.000000	0.000198
Max.	1.679803	0.310345	0.017750
SP			
Ave.	0.944524	0.947356	0.687560
SD	0.065266	0.111963	0.278814
Min.	0.797757	0.695715	0.242474
Max.	1.080351	1.278655	1.460767
HV			
Ave.	—	0.137784	0.296372
SD	—	0.117596	0.053300
Min.	—	—	0.043514
Max.	—	0.311075	0.327309

**Table 3:** Algorithm performance tested on ZDT3 problem.

Measure	VEPSO	VEPSOnds1	VEPSOnds2
NS			
Ave.	35.150000	99.600000	99.400000
SD	6.853997	3.405284	6.000000
Min.	21.000000	66.000000	40.000000
Max.	53.000000	100.000000	100.000000
GD			
Ave.	0.173060	0.009607	0.002040
SD	0.031253	0.008293	0.002268
Min.	0.079595	0.000433	0.000223
Max.	0.276801	0.039481	0.013231
SP			
Ave.	0.871146	1.109448	1.121149
SD	0.043319	0.086041	0.099980
Min.	0.701884	0.902861	0.858725
Max.	1.001428	1.322024	1.362217
HV			
Ave.	0.004722	0.373133	0.471686
SD	0.021699	0.083015	0.038568
Min.	—	0.112859	0.332399
Max.	0.167359	0.506222	0.514600

**Table 4:** Algorithm performance tested on ZDT4 problem.

Measure	VEPSO	VEPSOnds1	VEPSOnds2
NS			
Ave.	6.610000	95.250000	64.220000
SD	3.920665	16.518967	38.860949
Min.	1.000000	15.000000	4.000000
Max.	21.000000	100.000000	100.000000
GD			
Ave.	5.062543	0.383646	0.349438
SD	3.167428	0.478535	0.431632
Min.	0.000000	0.000155	0.000165
Max.	13.350278	2.049212	1.923652
SP			
Ave.	0.858655	1.035510	0.962023
SD	0.147255	0.347336	0.367664
Min.	0.483073	0.077112	0.144160
Max.	1.236461	1.419225	1.435101
HV			
Ave.	0.228824	0.399914	0.437755
SD	0.188151	0.159971	0.155761
Min.	—	—	—
Max.	0.573978	0.661941	0.660821

**Table 5:** Algorithm performance tested on ZDT6 problem.

Measure	VEPSO	VEPSOnds1	VEPSOnds2
NS			
Ave.	76.590000	78.040000	81.030000
SD	32.884891	26.684055	25.075021
Min.	11.000000	22.000000	24.000000
Max.	100.000000	100.000000	100.000000
GD			
Ave.	0.338537	0.260666	0.266259
SD	0.370336	0.158592	0.168404
Min.	0.001746	0.044137	0.035520
Max.	1.552521	0.709692	0.735990
SP			
Ave.	1.201796	1.276529	1.286909
SD	0.146782	0.083293	0.075052
Min.	0.492064	0.987981	1.067748
Max.	1.435395	1.437289	1.410091
HV			
Ave.	0.304584	0.303381	0.281256
SD	0.134813	0.102216	0.119017
Min.	—	0.038143	0.026496
Max.	0.400964	0.400780	0.401005

**Table 6:** Performance comparison based on ZDT1 test problem.

Measure	AbYSS	NSGA-II	SPEA2	SMPSO	VEPSOnds2
NS					
Ave.	100.000000	100.000000	100.000000	100.000000	99.790000
SD	0.000000	0.000000	0.000000	0.000000	1.458483
Min.	100.000000	100.000000	100.000000	100.000000	86.000000
Max.	100.000000	100.000000	100.000000	100.000000	100.000000
GD					
Ave.	0.000185	0.000223	0.000220	0.000117	0.002194
SD	0.000035	0.000038	0.000028	0.000031	0.003505
Min.	0.000125	0.000146	0.000154	0.000053	0.000169
Max.	0.000343	0.000374	0.000400	0.000172	0.019113
SP					
Ave.	0.105387	0.379129	0.148572	0.076608	0.571807
SD	0.012509	0.028973	0.012461	0.009200	0.248304
Min.	0.080690	0.282485	0.116765	0.056009	0.168144
Max.	0.136747	0.441002	0.174986	0.099653	1.127141
HV					
Ave.	0.661366	0.659333	0.659999	0.661801	0.631216
SD	0.000269	0.000301	0.000301	0.000100	0.046091
Min.	0.660267	0.658486	0.659347	0.661372	0.438793
Max.	0.661724	0.659909	0.660629	0.661991	0.661363

**Table 7:** Performance comparison based on ZDT2 test problem.

Measure	AbYSS	NSGA-II	SPEA2	SMPSO	VEPSOnds2
NS					
Ave.	100.000000	100.000000	100.000000	100.000000	97.490000
SD	0.000000	0.000000	0.000000	0.000000	7.832198
Min.	100.000000	100.000000	100.000000	100.000000	49.000000
Max.	100.000000	100.000000	100.000000	100.000000	100.000000
GD					
Ave.	0.000131	0.000176	0.000182	0.000051	0.002003
SD	0.000067	0.000066	0.000039	0.000003	0.003483
Min.	0.000056	0.000093	0.000090	0.000044	0.000198
Max.	0.000433	0.000707	0.000304	0.000060	0.017750
SP					
Ave.	0.130425	0.378029	0.158187	0.071698	0.687560
SD	0.090712	0.028949	0.027529	0.013981	0.278814
Min.	0.080831	0.311225	0.118114	0.035786	0.242474
Max.	0.833933	0.430516	0.365650	0.106749	1.460767
HV					
Ave.	0.325483	0.326117	0.326252	0.328576	0.296372
SD	0.023209	0.000297	0.000908	0.000077	0.053300
Min.	0.096409	0.325278	0.318785	0.328349	0.043514
Max.	0.328505	0.326696	0.327559	0.328736	0.327309

**Table 8:** Performance comparison based on ZDT3 test problem.

Measure	AbYSS	NSGA-II	SPEA2	SMPSO	VEPSOnds2
NS					
Ave.	100.000000	100.000000	100.000000	99.900000	99.400000
SD	0.000000	0.000000	0.000000	0.904534	6.000000
Min.	100.000000	100.000000	100.000000	91.000000	40.000000
Max.	100.000000	100.000000	100.000000	100.00000	100.000000
GD					
Ave.	0.000193	0.000211	0.000230	0.000203	0.002040
SD	0.000019	0.000013	0.000019	0.000061	0.002268
Min.	0.000144	0.000180	0.000184	0.000155	0.000223
Max.	0.000264	0.000268	0.000327	0.000717	0.013231
SP					
Ave.	0.707651	0.747853	0.711165	0.717493	1.121149
SD	0.013739	0.015736	0.008840	0.032822	0.099980
Min.	0.696859	0.715199	0.698590	0.697943	0.858725
Max.	0.796404	0.793183	0.775317	0.950901	1.362217
HV					
Ave.	0.512386	0.514813	0.513996	0.514996	0.471686
SD	0.011314	0.000159	0.000675	0.001737	0.038568
Min.	0.463776	0.514449	0.510764	0.500484	0.332399
Max.	0.515960	0.515185	0.514668	0.515818	0.514600

**Table 9:** Performance comparison based on ZDT4 test problem.

Measure	AbYSS	NSGA-II	SPEA2	SMPSO	VEPSOnds2
NS					
Ave.	99.680000	100.000000	100.000000	100.000000	64.220000
SD	3.100603	0.000000	0.000000	0.000000	38.860949
Min.	69.000000	100.000000	100.000000	100.000000	4.000000
Max.	100.000000	100.000000	100.000000	100.000000	100.000000
GD					
Ave.	0.001231	0.000486	0.000923	0.0001347	0.349438
SD	0.002632	0.000235	0.001428	0.000027	0.431632
Min.	0.000148	0.000163	0.000176	0.000070	0.000165
Max.	0.014472	0.001374	0.012292	0.000187	1.923652
SP					
Ave.	0.159842	0.392885	0.298269	0.092281	0.962023
SD	0.120180	0.037083	0.125809	0.011777	0.367664
Min.	0.078244	0.324860	0.137934	0.067379	0.144160
Max.	1.073669	0.473358	0.884091	0.124253	1.435101
HV					
Ave.	0.646058	0.654655	0.645336	0.661401	0.437755
SD	0.034449	0.003406	0.018773	0.000162	0.155761
Min.	0.472299	0.642177	0.505799	0.660934	0.000000
Max.	0.661594	0.659710	0.658784	0.661726	0.660821

**Table 10:** Performance comparison based on ZDT6 test problem.

Measure	AbYSS	NSGA-II	SPEA2	SMPSO	VEPSOnds2
NS					
Ave.	100.000000	100.000000	100.000000	100.000000	81.030000
SD	0.000000	0.000000	0.000000	0.000000	25.075021
Min.	100.000000	100.000000	100.000000	100.000000	24.000000
Max.	100.000000	100.000000	100.000000	100.000000	100.000000
GD					
Ave.	0.000549	0.001034	0.001761	0.012853	0.266259
SD	0.000015	0.000102	0.000192	0.024813	0.168404
Min.	0.000510	0.000804	0.001267	0.000502	0.035520
Max.	0.000596	0.001360	0.002207	0.092434	0.735990
SP					
Ave.	0.097740	0.357160	0.226433	0.390481	1.286909
SD	0.013129	0.031711	0.020658	0.497140	0.075052
Min.	0.070455	0.282201	0.179482	0.042666	1.067748
Max.	0.130389	0.441311	0.292897	1.377582	1.410091
HV					
Ave.	0.400346	0.388304	0.378377	0.401280	0.281256
SD	0.000172	0.001604	0.002714	0.000076	0.119017
Min.	0.399821	0.383637	0.371907	0.401081	0.026496
Max.	0.400842	0.392123	0.385626	0.401402	0.401005
